# Cross-sectional brain volumetric measures and disability outcomes in relapsing–remitting multiple sclerosis

**DOI:** 10.1007/s00415-026-13788-z

**Published:** 2026-04-01

**Authors:** Michael T. G. Hayes, Heidi N. Beadnall, Daniel Merlo, Mastura Monif, Chao Zhu, Michael Barnett, Katherine Buzzard, Dana Horakova, Tomas Kalincik, Guy Laureys, Elaine Lui, Jeannette Lechner-Scott, Ai-Lan Nguyen, Francesco Patti, Diana M. Sima, Dirk Smeets, Manuela Vaneckova, Wim Van Hecke, Vincent Van Pesch, Anneke van der Walt, Helmut Butzkueven

**Affiliations:** 1 Department of Neuroscience, School of Translational Medicine, Faculty of Medicine, Nursing and Health Sciences, Monash University, The Alfred Centre, Level 6, 99 Commercial Road, Melbourne, VIC 3004 Australia; 2 Department of Neurology, Alfred Health, Melbourne, VIC 3004 Australia; 3 Brain and Mind Centre, The University of Sydney, Sydney, NSW 2050 Australia; 4 Department of Neurology, Royal Prince Alfred Hospital, Sydney, NSW 2050 Australia; 5 Department of Neurology, Box Hill Hospital, Box Hill, VIC 3128 Australia; 6 Department of Neurosciences, Eastern Health Clinical School, Monash University, Box Hill, VIC 3128 Australia; 7 Department of Neurology and Centre of Clinical Neuroscience, First Faculty of Medicine, Charles University and General University Hospital, 121 08 Prague, Czech Republic; 8 Clinical Outcomes Research Unit (CORe), Department of Medicine, University of Melbourne, Melbourne, VIC 3010 Australia; 9 Neuroimmunology Centre, Royal Melbourne Hospital, Melbourne, VIC 3000 Australia; 10 Department of Neurology, Ghent University Hospital, 9000 Ghent, Belgium; 11 Department of Medical Imaging, Royal Melbourne Hospital, Melbourne, VIC 3050 Australia; 12 Department of Radiology, University of Melbourne, Melbourne, VIC 3010 Australia; 13 University of Newcastle, Newcastle, NSW 2308 Australia; 14 Department of Neurology, John Hunter Hospital, Newcastle, NSW 2305 Australia; 15 Department of Medical and Surgical Sciences and Advanced Technologies “GF Ingrassia”, University of Catania, 95124 Catania, Italy; 16 Azienda Ospedaliero-Universitaria Policlinico “G. Rodolico-San Marco”, 95123 Catania, Italy; 17 icometrix, 3012 Louvain, Belgium; 18 Department of Radiology, First Faculty of Medicine, Charles University and General University Hospital, 121 08 Prague, Czech Republic; 19 Department of Laboratory Medicine, Cliniques Universitaires Saint-Luc, 1200 Brussels, Belgium; 20 Neurochemistry Unit, Institute of Neuroscience, Université Catholique de Louvain, 1348 Louvain-la-Neuve, Belgium

**Keywords:** Multiple sclerosis, MRI, Brain atrophy, Lateral ventricle volume, Prognosis

## Abstract

**Background:**

Automated cross-sectional magnetic resonance imaging (MRI) measures of brain volume are becoming available in clinical practice; however, their prognostic utility in multiple sclerosis remains unclear.

**Objectives:**

We investigated associations between cross-sectional brain and brain lesion volume measures and subsequent disability outcomes in relapsing–remitting multiple sclerosis (RRMS).

**Methods:**

We conducted a multicentre international cohort study using data collected prospectively in the MSBase Registry. MRI analysis was performed using icobrain ms. Associations between MRI volumetric measurements and confirmed disability worsening (CDW), progression independent of relapse activity (PIRA), secondary progressive multiple sclerosis (SPMS) and confirmed disability improvement (CDI) were examined using multivariable Cox proportional hazards models.

**Results:**

A total of 1598 patients were included and 68.6% received high-efficacy disease-modifying therapy during follow-up. Normalised lateral ventricular volume (mL) was significantly associated with all disability outcomes, including PIRA (hazard ratio 1.007; 95% confidence interval 1.002 − 1.012; *p* = 0.009). Higher normalised whole brain, grey matter and white matter volumes were all significantly associated with a lower hazard of CDW and higher hazard of achieving CDI. Higher normalised white matter volume was also significantly associated with a lower hazard of PIRA. Higher T1-weighted black hole lesion volume was significantly associated with an increased hazard of conversion to SPMS; lesion volume measures were otherwise not significantly associated with disability outcomes.

**Conclusions:**

Automated MRI measures of brain volume from a single time point demonstrated prognostic utility in a highly treated cohort of people with RRMS. The results suggest that normalised lateral ventricular volume is a particularly informative prognostic marker across multiple disability outcomes.

**Supplementary Information:**

The online version contains supplementary material available at 10.1007/s00415-026-13788-z.

## Introduction

In routine multiple sclerosis (MS) care, use of magnetic resonance imaging (MRI) remains limited to visual lesion surveillance. However, conventional lesion metrics show modest associations with disability outcomes—a phenomenon termed the “clinical-MRI paradox” [1, 2]—which can be explained by the observation that focal inflammatory activity is not the only pathophysiological process that contributes to the risk of disability accrual [3]. Clinically translatable MRI biomarkers of other key processes, including chronic inflammation and neuroaxonal loss, have the potential to drastically improve MS prognostication and facilitate timely, personalised treatment decisions for people with MS (PwMS).

Certain MRI markers of brain atrophy are known to be associated with MS outcomes [4–7]. However, their clinical implementation has been hindered by technical and physiological factors that limit their reliability in the clinical setting [8]. Longitudinal measures have the advantage of being dynamic markers, but face more sources of variability, including inter- and intra-scanner variability [9, 10]. Cross-sectional MRI volumetric measures are technically much easier to obtain and could provide prognostic information from a single time point, without the need to wait 12 months or longer for follow-up data. While cross-sectional measures have been associated with clinical outcomes, including the Expanded Disability Status Scale (EDSS), most studies used single EDSS follow-up scores [4, 5], rather than confirmed EDSS worsening—a more accurate measure of irreversible disability progression [11]—and did not involve cohorts treated with high-efficacy disease-modifying therapies (HET). Furthermore, few studies have explored the association between cross-sectional brain volumetric measures and confirmed disability improvement (CDI) or progression independent of relapse activity (PIRA) [12], two important and relatively novel outcome measures. PIRA refers to insidious disability accrual that occurs without clinically evident relapses, and accounts for approximately 50% of all disability accrual events in relapsing–remitting MS (RRMS) [13, 14]. This form of progression is often initially overlooked in the clinical setting [15]; establishing MRI predictors of PIRA would lead to earlier recognition and intervention.

The aim of our study was to identify associations between cross-sectional brain volumetric measurements, taken from routine clinical MRI scans, and long-term disability outcomes in people with RRMS. These outcomes were (1) sustained confirmed disability worsening (CDW), (2) sustained CDI, (3) sustained PIRA and (4) conversion to secondary progressive multiple sclerosis (SPMS). The MRI measures investigated were (1) normalised whole brain volume (WBV), (2) normalised grey matter volume (GMV), (3) normalised white matter volume (WMV), (4) normalised lateral ventricular volume (LVV), (5) T1-weighted black hole lesion volume (BHLV) and (6) T2-weighted fluid-attenuated inversion recovery lesion volume (T2LV).

## Methods

### Study design

**Fig. 1:**
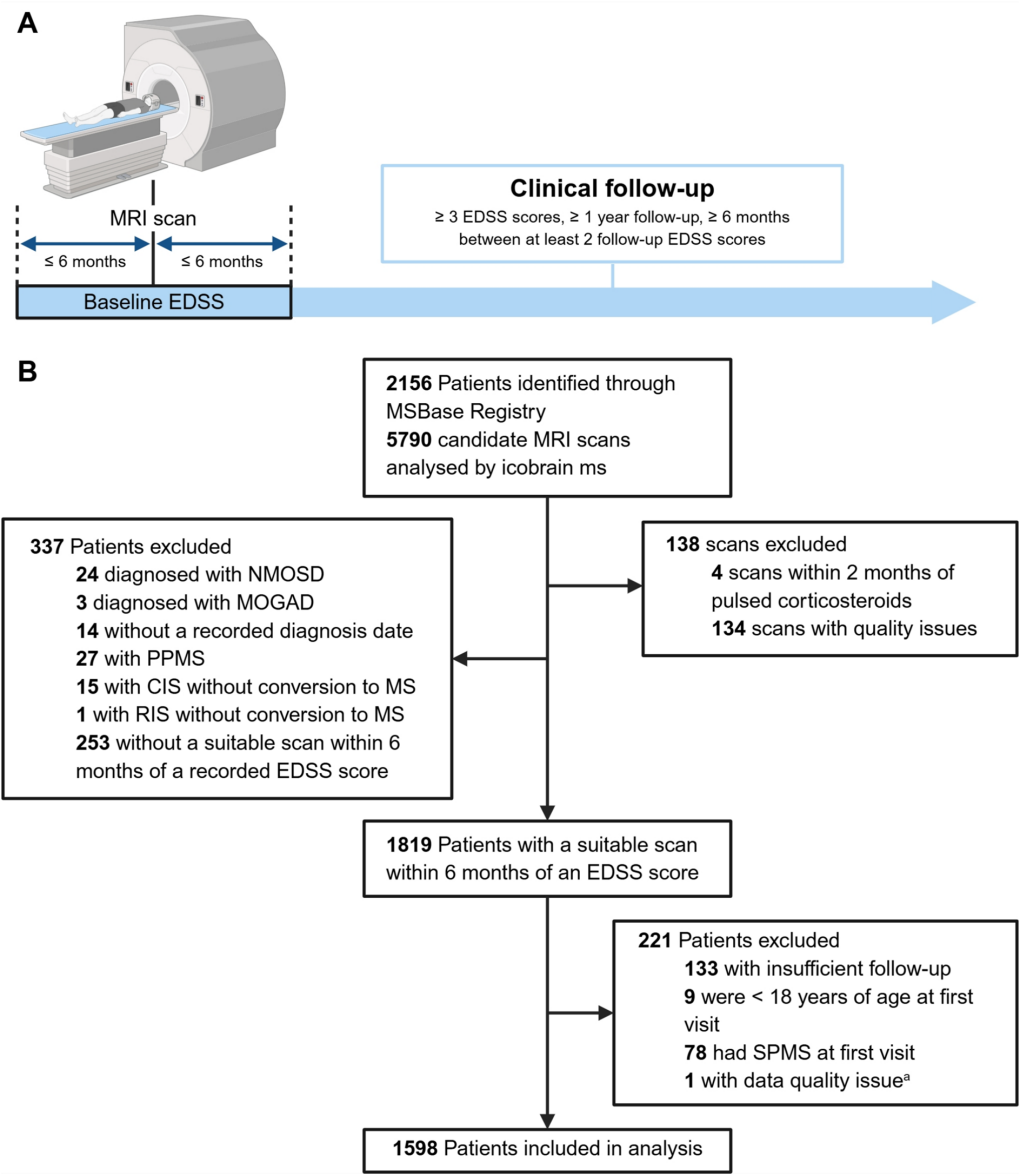
**A** Study design—MRI acquisition and clinical data collection. **B** Study population flow chart. *CIS* clinically isolated syndrome, *EDSS* Expanded Disability Status Scale, *MOGAD* myelin oligodendrocyte glycoprotein antibody-associated disease, *MRI* magnetic resonance imaging, *MS* multiple sclerosis, *NMOSD* neuromyelitis optica spectrum disorder, *PPMS* primary progressive multiple sclerosis, *RIS* radiologically isolated syndrome, *SPMS* secondary progressive multiple sclerosis. ^a^Anomalous sequence of EDSS scores deemed unrelated to MS. Created in BioRender: BioRender.com/yg3k2iq

This was a retrospective cohort study that utilised data collected prospectively from participants in the MSBase Registry [16] who underwent MRI as part of routine clinical care at nine MS centres in Australia, Belgium, Czech Republic and Italy. Participants were eligible for inclusion if they had RRMS, a suitable MRI scan performed within 6 months of an EDSS score, two or more follow-up EDSS scores over at least 1 year and were 18 years of age or older at baseline visit. The baseline EDSS score was the score recorded closest to the scan date and no more than 6 months before or after the scan (Fig. 1A). Scores recorded within 30 days of a relapse were excluded from use as baseline scores. PwMS with a baseline diagnosis of clinically isolated syndrome (CIS) who converted to RRMS during follow-up were included, whereas those with SPMS at baseline visit were excluded.

Participants’ demographic and clinical details were sourced from the MSBase Registry, including date of MS symptom onset (used to calculate disease duration at baseline), EDSS scores, relapse dates and disease-modifying therapy (DMT) received. EDSS was scored by accredited practitioners (Neurostatus-certified) at each centre. DMTs were grouped into two categories for analysis: (1) HET: cladribine, natalizumab, ocrelizumab, ofatumumab, rituximab, alemtuzumab or autologous haematopoietic stem cell transplant (AHSCT), and (2) all other DMTs or no DMT. DMTs received during the study period and thresholds used to calculate treatment intervals are summarised in Online Supplementary Table 1.

The MSBase Registry (World Health Organization International Clinical Trials Registry Platform ID: ACTRN12605000455662) was approved by the Alfred Health Human Research Ethics Committee and by the local ethics committees in all participating centres. All participants provided written informed consent prior to their inclusion in the study. The study followed the Strengthening the Reporting of Observational Studies in Epidemiology (STROBE) reporting guideline for cohort studies.

### MRI analysis

MRI scans were analysed using icobrain ms version 3.1 [17] for eight participating centres (*n* = 1402) and icobrain ms version 5.13 [18] for one centre (*n* = 196) due to software upgrades between analyses. Cross-sectional volumetric measurements, grouped by icobrain ms version, are displayed in Online Supplementary Fig. 1. The requirements for analysis were a 2D or 3D T2-weighted fluid-attenuated inversion recovery sequence with a maximum slice thickness of 3 mm, and a 3D T1-weighted sequence with a maximum slice thickness of 1.6 mm, acquired with a 1.5 or 3 Tesla scanner during the same session. Scan dates ranged from June 2008 to February 2024. When multiple scans were available for a participant, the earliest was used. Scans performed within 2 months of pulsed, high-dose corticosteroids were excluded, due to the potential for this treatment to cause transient reductions in brain volume [19]. A detailed description of the icobrain ms analysis pipelines and a list of MRI scanners used (Supplementary Table 2) is provided in the Online Supplementary Material. The icobrain ms pipelines incorporate automated quality control processes that flag data for manual review by an experienced rater. Each scan is assigned a “quality control status”, either (1) approved, (2) approved with remarks, (3) rejected or (4) unusable. Scans were included in the study if they were approved without remarks or with remarks indicating minor issues unlikely to impact volumetric measurements. All MRI measurements are presented in millilitres (mL).

### Study outcomes

Four disability-related outcomes were measured. Sustained CDW was defined as an EDSS increase of ≥ 1.5, ≥ 1.0 or ≥ 0.5 if the baseline score was 0, 1.0–5.5 or ≥ 6.0, respectively, confirmed over 6 months and sustained through follow-up [11]. Sustained PIRA required the same EDSS increase, but without relapses between the preceding score and first score representing disability worsening [20, 21]. For both CDW and PIRA, EDSS scores recorded less than 30 days after a relapse were ineligible for use as confirmatory scores. SPMS was a data-driven diagnosis based on the Lorscheider et al. [22] criteria (d-SPMS), which requires an EDSS increase of ≥ 1.0 or ≥ 0.5 if the baseline score is ≤ 5.5 or ≥ 6.0, respectively, with a post-worsening EDSS score ≥ 4 and a pyramidal Functional Systems score ≥ 2, without a preceding relapse, confirmed over 3 months. When identifying PIRA and d-SPMS, the baseline EDSS score was reset to the first score recorded more than 30 days after any relapse. Sustained CDI required a decrease in EDSS of ≥ 1.0 or ≥ 0.5 if the baseline score was 2.0–6.0 or ≥ 6.5, respectively, confirmed over 6 months and sustained through follow-up. Only participants with a baseline EDSS ≥ 2 were included in the CDI analysis, as scores below this threshold represent minimal neurological deficits [23]. Outcome dates were defined by the first associated EDSS change.

### Statistical analysis

Participant characteristics were reported as mean (standard deviation, SD) for normally distributed continuous variables, median (interquartile range, IQR) for non-normal continuous variables and frequency and percentage for categorical variables. Associations between MRI metrics and CDW, PIRA, d-SPMS and CDI were examined using multivariable Cox proportional hazards models. The covariates were chosen a priori and included age, disease duration and EDSS at baseline, sex, number of relapses within the 2-year period before baseline, proportion of time on DMT from diagnosis to baseline, icobrain ms software version and DMT during follow-up as a time-varying covariate. Models featuring either (1) single MRI metrics, (2) WBV, GMV, WMV or LVV and BHLV or (3) T2LV and WBV were run to assess independent relationships between each metric and disability outcomes. WBV, GMV, WMV and LVV were analysed in separate models due to collinearity, as were BHLV and T2LV. BHLV, rather than T2LV, was included as a covariate for brain volumetric measures due to its stronger associations with disability outcomes [24–26].

To assess the potential impact of heterogeneity across study sites, particularly in MRI acquisition, a sensitivity analysis was performed in which Cox regression models were stratified by study site. To evaluate the potential influence of pseudoatrophy associated with recent initiation of DMT [8], a second sensitivity analysis was conducted in which participants were excluded from Cox regression analyses if they had started their first DMT within the 6 months preceding MRI or recommenced DMT within the 6 months preceding MRI following a treatment-free period of 6 months or more.

The effect of HET on relapse rate during follow-up compared with other/no DMT was examined using mixed-effects negative binomial regression, with participant ID as a random effect and the following variables as fixed effects: age, disease duration and EDSS at baseline, sex, number of relapses within the 2-year period before baseline and proportion of time on DMT from diagnosis to baseline. Statistical analysis was performed with R software, version 4.4.0, and statistical significance reported at *p* < 0.05.

## Results

In total, 1598 PwMS met inclusion criteria (Fig. 1B); 891 had a baseline EDSS score ≥ 2, and 1190 (74.5%) were female. Clinical and MRI characteristics are summarised in Table 1. The mean age at baseline was 40.2 years (SD 10.7) and the median disease duration was 7.6 years (IQR 3.8–13.3). The median follow-up was 7.8 years (IQR 4.9–9.6), during which time 1096 participants (68.6%) received at least one HET. The number of participants who experienced CDW, PIRA, d-SPMS and CDI was 371, 248, 169 and 107, respectively. Standardised mean differences for each characteristic compared between groups with and without each disability outcome are reported in Online Supplementary Tables 3–6.

**Table 1:** Clinical and MRI characteristics of the whole cohort and each disability outcome group

Characteristic	Whole cohort *n* = 1598	CDW *n* = 371 (23.2%)	PIRA *n* = 248 (15.5%)	d-SPMS *n* = 169 (10.6%)	CDI *n* = 107 (12.0%)^a^
Age at baseline (years), mean (SD)	40.2 (10.7)	42.2 (11.0)	44.5 (11.3)	45.1 (11.0)	38.9 (10.0)
Female, *n* (%)	1190 (74.5)	277 (74.7)	178 (71.8)	120 (71.0)	84 (78.5)
Disease duration at baseline (years), median (IQR)	7.6 (3.8 − 13.3)	9.4 (5.3 − 14.7)	10.0 (5.6 − 15.1)	11.3 (7.1 − 17.0)	5.8 (1.9 − 13.4)
Number of relapses within 2 years of baseline, median (IQR)	0 (0 − 1)	0 (0 − 2)	0 (0 − 1)	1 (0 − 2)	1 (0 − 2)
CIS at baseline, n (%)	47 (2.9)	11 (3.0)	5 (2.0)	3 (1.8)	6 (5.6)
Time from first DMT initiation to baseline (years), median (IQR)	5.1 (2.2 − 9.1)	5.9 (3.0 − 9.6)	6.0 (3.1 − 10.4)	7.2 (3.8 − 11.2)	4.8 (1.7 − 7.6)
No DMT use before baseline, *n* (%)	160 (10.0)	27 (7.3)	18 (7.3)	10 (5.9)	27 (25.2)
Taking any DMT at baseline, *n* (%)	1371 (85.8)	334 (90.0)	220 (88.7)	154 (91.1)	74 (69.2)
Taking HET at baseline, *n* (%)	533 (33.4)	120 (32.4)	91 (36.7)	68 (40.2)	25 (30.5)
Took ≥ 1 HET during follow-up, *n* (%)	1096 (68.6)	271 (73.1)	172 (69.4)	134 (79.3)	80 (74.8)
EDSS score at baseline, median (IQR)	2 (1 − 3)	2 (1 − 3.5)	2 (1 − 4)	3.5 (2.5 − 4.5)	3 (2 − 4)
EDSS score at final follow-up, median (IQR)	2 (1.5 − 3.5)	4 (2.5 − 6)	4.5 (2.5 − 6)	6 (5 − 6.5)	1 (1.5 − 2)
Time to disability outcome (years), median (IQR)	NA	4.0 (2.1 − 6.6)	3.8 (2.0 − 6.2)	4.7 (2.5 − 7.2)	1.6 (0.8 − 3.5)
Duration of follow-up (years), median (IQR)	7.8 (4.9 − 9.6)	8.8 (6.8 − 10.2)	8.2 (6.3 − 9.7)	8.5 (6.8 − 10.1)	8.1 (5.4 − 9.7)
WBV (mL), mean (SD)	1534.5 (71.6)	1514.6 (69.7)	1510.9 (72.3)	1497.3 (71.5)	1559.8 (67.5)
GMV (mL), mean (SD)	910.2 (53.7)	897.3 (50.2)	896.1 (51.4)	887.6 (48.4)	923.8 (53.0)
WMV (mL), mean (SD)	624.3 (42.3)	617.3 (44.1)	614.8 (46.7)	609.8 (47.6)	636.0 (38.5)
LVV (mL), median (IQR)	28.0 (20.0 − 39.0)	30.0 (23.0 − 43.0)	32.0 (25.0 − 45.3)	36.0 (27.0 − 52.0)	25.0 (18.0 − 38.0)
BHLV (mL), median (IQR)	2.2 (0.9 − 5.1)	2.7 (1.2 − 5.5)	2.9 (1.4 − 6.5)	4.0 (1.8 − 8.5)	2.1 (0.6 − 5.3)
T2LV (mL), median (IQR)	3.8 (1.5 − 8.3)	4.8 (2.3 − 9.4)	5.0 (2.4 − 9.7)	6.2 (2.9 − 13.8)	3.4 (1.1 − 7.7)

*BHLV* black hole lesion volume, *CDI* confirmed disability improvement, *CDW* confirmed disability worsening, *CIS* clinically isolated syndrome, *DMT* disease-modifying therapy, *EDSS* Expanded Disability Status Scale, *GMV* normalised grey matter volume, *HET* high-efficacy disease-modifying therapy, *LVV* normalised lateral ventricular volume, *PIRA* progression independent of relapse activity, *d-SPMS* data-driven secondary progressive multiple sclerosis, *T2LV* T2-weighted fluid-attenuated inversion recovery lesion volume, *WBV* normalised whole brain volume, *WMV* normalised white matter volume

^a^The 891 participants with a baseline EDSS score ≥ 2 were included in CDI analyses

Participants with CDW, PIRA and d-SPMS were older, had a longer disease duration and higher baseline EDSS compared to those without the respective outcome. Participants with CDI were younger, had a shorter disease duration and were less likely to have received any DMT or HET at baseline compared to those without CDI. WBV, GMV and WMV were lower in participants who developed CDW, PIRA and d-SPMS and higher in participants who experienced CDI compared to those without the respective outcome. The opposite was found for LVV, BHLV and T2LV, which were all higher in participants who developed CDW, PIRA and d-SPMS and lower in participants who experienced CDI (Online Supplementary Tables 3–6).

### MRI measures as predictors of disability outcomes

In multivariable Cox proportional hazards models adjusted for BHLV, WBV was significantly associated with CDW and CDI (Table 2). The hazard ratio (HR) was 0.997 for CDW (95% confidence interval (CI) 0.995 − 0.998; *p* < 0.001) and 1.008 for CDI (95% CI 1.005 − 1.012; *p* < 0.001), equating to a 0.3% reduction in the hazard of CDW and 0.8% increase in the hazard of achieving CDI for every 1 mL increase in WBV. In models that did not include BHLV, WBV was significantly associated with PIRA and d-SPMS (Online Supplementary Table 7), but the association was no longer significant after adjustment for BHLV.

**Table 2:** Multivariable Cox regression analyses of associations between brain volumetric measures and disability outcomes

MRI predictor	CDW HR (95% CI)	PIRA HR (95% CI)	d-SPMS HR (95% CI)	CDI HR (95% CI)
WBV, mL^a^	0.997 (0.995 − 0.998), ***p* < 0.001**	0.998 (0.996 − 1.000), *p* = 0.13	0.998 (0.996 − 1.001), *p* = 0.16	1.008 (1.005 − 1.012), ***p* < 0.001**
GMV, mL^a^	0.997 (0.995 − 0.9996), ***p* = 0.02**	1.001 (0.998 − 1.004), *p* = 0.72	1.000 (0.996 − 1.004), *p* = 0.99	1.009 (1.004 − 1.013), ***p* < 0.001**
WMV, mL^a^	0.996 (0.993 − 0.998), ***p* = 0.002**	0.996 (0.993 − 0.999), ***p* = 0.02**	0.996 (0.992 − 1.000) *p* = 0.06	1.007 (1.003 − 1.012), ***p* = 0.002**
LVV, mL^a^	1.008 (1.003 − 1.012), ***p* < 0.001**	1.007 (1.002 − 1.012), ***p* = 0.009**	1.009 (1.002 − 1.015), ***p* = 0.007**	0.987 (0.975 − 0.999), ***p* = 0.04**
BHLV, mL^b^	1.014 (0.988 − 1.040), *p* = 0.30	1.026 (0.996 − 1.057), *p* = 0.09	1.032 (1.001 − 1.064), ***p* = 0.04**	1.010 (0.967 − 1.056), *p* = 0.65
T2LV, mL^b^	1.013 (0.994 − 1.031), *p* = 0.19	1.020 (0.998 − 1.042), *p* = 0.08	1.023 (0.999 − 1.048), *p* = 0.06	0.991 (0.958 − 1.026), *p* = 0.62

Bold values indicate statistically significant results

*BHLV* black hole lesion volume, *CDI* confirmed disability improvement, *CDW* confirmed disability worsening, *GMV* normalised grey matter volume, *HR* hazard ratio, *LVV* normalised lateral ventricular volume, *MRI* magnetic resonance imaging, *PIRA* progression independent of relapse activity, *d-SPMS* data-driven secondary progressive multiple sclerosis, *T2LV* T2-weighted fluid-attenuated inversion recovery lesion volume, *WBV* normalised whole brain volume, *WMV* normalised white matter volume

^a^BHLV was included as a covariate alongside age, disease duration and EDSS at baseline, number of relapses in the 2-year period preceding baseline, proportion of time on DMT pre-baseline, sex, icobrain ms software version and DMT as a time-varying covariate

^b^WBV was included as a covariate alongside age, disease duration and EDSS at baseline, number of relapses in the 2-year period preceding baseline, proportion of time on DMT pre-baseline, sex, icobrain ms software version and DMT as a time-varying covariate

GMV was significantly associated with CDW (HR 0.997; 95% CI 0.995 − 0.9996; *p* = 0.02) and CDI (HR 1.009; 95% CI 1.004 − 1.013; *p* < 0.001) in multivariable models adjusted for BHLV. However, GMV was not significantly associated with PIRA or d-SPMS, irrespective of whether BHLV was included in the models. In contrast, WMV was significantly associated with PIRA (HR 0.996; 95% CI 0.993 − 0.999; *p* = 0.02) in multivariable models adjusted for BHLV, while also being significantly associated with CDW (HR 0.996; 95% CI 0.993 − 0.998; *p* = 0.002) and CDI (HR 1.007; 95% CI 1.003 − 1.012; *p* = 0.002). The association between WMV and d-SPMS was not statistically significant when adjusted for BHLV (HR 0.996; 95% CI 0.992 − 1.000, *p* = 0.06), but became significant when BHLV was excluded from the model (Online Supplementary Table 7).

In Cox regression models adjusted for BHLV, LVV was significantly associated with each of the four clinical outcomes, with a HR of 1.008 (95% CI 1.003 − 1.012; *p* < 0.001) for CDW, 1.007 (95% CI 1.002 − 1.012; *p* = 0.009) for PIRA, 1.009 (95% CI 1.002 − 1.015; *p* = 0.007) for d-SPMS and 0.987 (95% CI 0.975 − 0.999; *p* = 0.04) for CDI. Among lesion volume measures, the only significant association in models adjusted for WBV was between BHLV and d-SPMS (HR 1.032; 95% CI 1.001 − 1.064; *p* = 0.04), equating to a 3.2% increase in the hazard of d-SPMS per 1 mL increase in BHLV.

In their respective Cox regression models, WBV, GMV and LVV showed the strongest associations with CDW among the covariates, based on having the largest Wald statistics. WMV showed the second strongest association with CDW after older age at baseline (Wald statistic 9.7; *p* = 0.002 for WMV vs 14.9; *p* < 0.001 for age at baseline). Across all Cox regression models, older age at baseline showed the strongest association with PIRA, while WMV and LVV ranked second in their respective models. Higher baseline EDSS was most strongly associated with d-SPMS in all Cox regression models, followed by age at baseline. In the model including LVV and BHLV, LVV and BHLV ranked third and fourth, respectively, based on the Wald statistic for association with d-SPMS (6.5; *p* = 0.01 for LVV vs 3.0; *p* = 0.08 for BHLV). WBV, GMV and WMV had larger Wald statistics for association with CDI than either age or MS duration at baseline. However, shorter MS duration at baseline showed a stronger association with CDI than LVV (Wald statistic 10.1; *p* = 0.002 for MS duration at baseline vs 5.1; *p* = 0.02 for LVV). Sex was not significantly associated with any of the four disability outcomes.

**Fig. 2:**
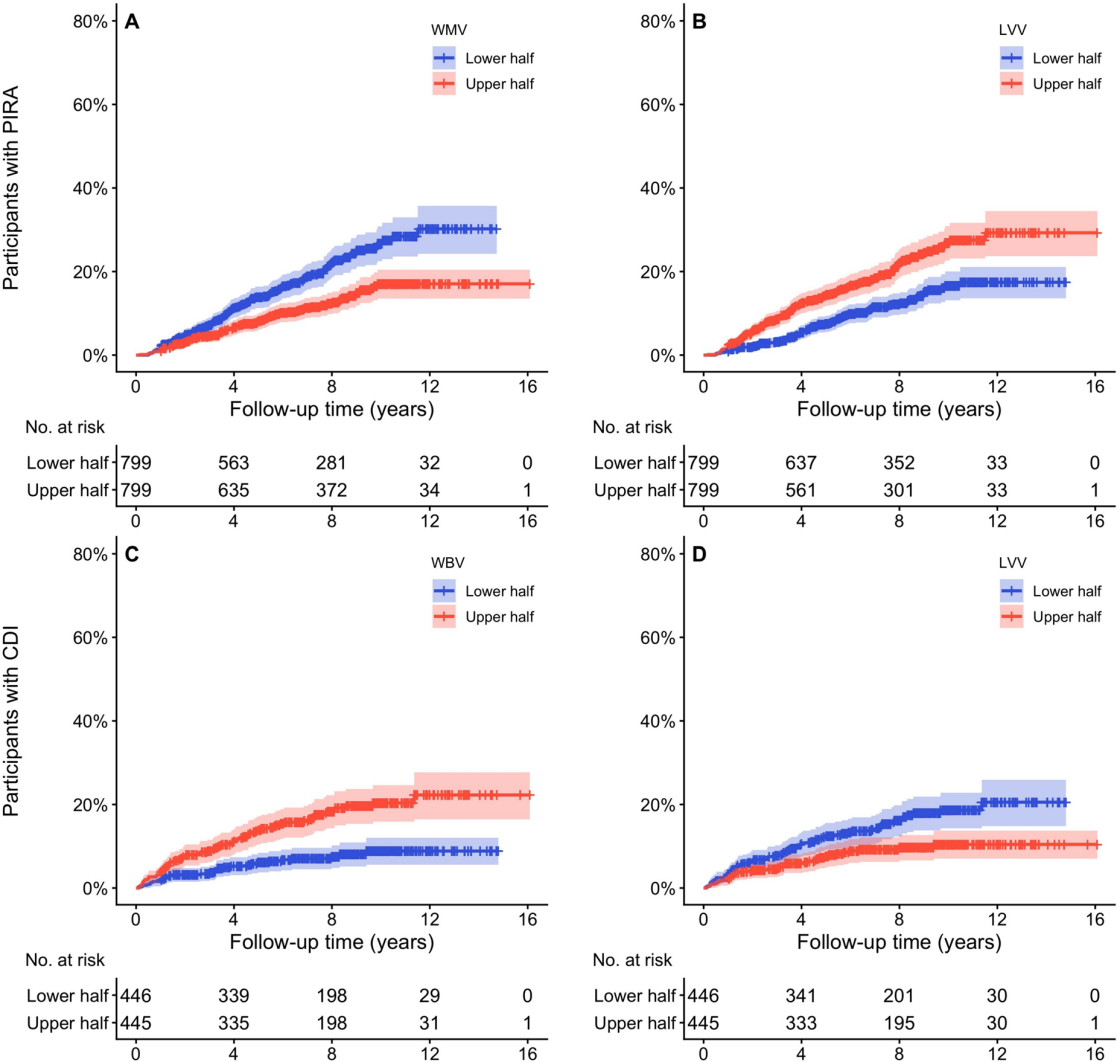
Survival curves for time to PIRA and time to CDI. **A** Time to PIRA based on WMV, **B** time to PIRA based on LVV, **C** time to CDI based on WBV and **D** time to CDI based on LVV. The red curve represents the upper half and the blue curve represents the lower half of the cohort for the associated brain MRI volumetric measurement, with the lighter shade reflecting the 95% CI. *CDI* confirmed disability improvement, *LVV* normalised lateral ventricular volume, *PIRA* progression independent of relapse activity, *WBV* normalised whole brain volume, *WMV* normalised white matter volume

To complement the multivariable Cox regression results, selected key associations between MRI metrics and disability outcomes are illustrated in Fig. 2 as unadjusted Kaplan–Meier survival curves, with the cohort stratified into upper and lower halves according to the associated brain volumetric measure. Survival curves for the remaining MRI measures and outcomes are illustrated in Online Supplementary Fig. 2–5.

### Sensitivity analyses

In the sensitivity analysis stratified by study site (Online Supplementary Table 8), the results were predominantly consistent with the primary analysis. The associations between GMV and CDW (HR 0.998; 95% CI 0.996 − 1.001; *p* = 0.2), and between WMV and PIRA (HR 0.997; 95% CI 0.993 − 1.000; *p* = 0.08) were attenuated and no longer statistically significant, but the direction of effect remained unchanged.

Of the 1598 participants, 83 commenced their first DMT within 6 months of the MRI scan and 27 initiated DMT within 6 months of the scan following a treatment gap of at least 6 months. In the sensitivity analysis excluding these participants (Online Supplementary Table 9), the results were also largely consistent with the primary analysis. The association between LVV and CDI was the only result that was attenuated and no longer statistically significant (HR 0.989; 95% CI 0.976 − 1.003, *p* = 0.11).

### Disease-modifying therapies and relapse rate

HET significantly reduced relapse rate during follow-up compared to other/no DMT in the mixed-effects negative binomial regression model. The incidence rate ratio was 0.31 (95% CI 0.27 − 0.36; *p* < 0.001), equating to a 69% reduction in relapse rate.

## Discussion

This large longitudinal cohort study demonstrated that certain cross-sectional brain MRI volumetric measures are associated with subsequent sustained disability events in people with RRMS in the era of HET. Higher WBV was independently associated with a lower hazard of CDW during follow-up. While WBV was significantly associated with PIRA and d-SPMS in Cox proportional hazards models without BHLV as a covariate, this was not the case after adjustment for BHLV. Many studies have investigated associations between cross-sectional WBV measures and MS outcomes, with mixed results [27]. Most showed correlations between WBV and both baseline and single follow-up EDSS scores [27], but when CDW was used, the results were inconsistent [28, 29]. A study using standardised, single-scanner MRI found that baseline grey matter fraction, but not brain parenchymal fraction (a measure of WBV), predicted 3-month CDW over 13-year follow-up [29]. In our study, lower GMV was also significantly associated with an increased hazard of CDW.

Interestingly, we found a significant association between higher cross-sectional WMV and a lower hazard of subsequent PIRA, while neither WBV nor GMV were significantly associated with PIRA. To our knowledge, this is the first study to report on the relationship between cross-sectional WMV and PIRA. Longitudinal studies in MS have generally reported higher rates of grey matter atrophy and stronger associations between grey matter atrophy and disability progression, including PIRA [7], than for white matter atrophy [5, 6, 8, 30–32]. However, microstructural damage in key white matter regions has been found to be associated with PIRA [33]. Lower WMV may indicate greater axonal loss [34] and functional disconnection between grey matter structures [30], and may be associated with secondary grey matter atrophy due to Wallerian degeneration [31]. Thus, it is biologically plausible that lower cross-sectional WMV reflects a state of reduced neurological reserve and greater susceptibility to accelerated neuroaxonal loss and functional decompensation, associated with a higher risk of PIRA.

Importantly, we found that larger LVV was independently associated with a higher hazard of all disability worsening outcomes. Progressive enlargement of the lateral ventricles is known to be associated with worse clinical outcomes, including CDW [4, 6, 35]. However, there has been limited evidence to support cross-sectional LVV as a predictor of disability outcomes. Seminal studies that found associations between disability outcomes and progressive ventricular enlargement—but not baseline ventricular volume—involved cohorts with CIS [35] or early RRMS [6]. Our cohort was larger and had a mean disease duration of 7.6 years at baseline, enabling more time for ventricular enlargement to occur before the study MRI, which may partly explain why cross-sectional LVV was a significant predictor of disability outcomes in our study, but not earlier studies [6, 35].

LVV is a proxy measure for brain atrophy and may be the most robust marker of neuroaxonal loss in the clinical setting [6, 36]. It is less sensitive to scan quality issues, including incomplete head coverage, gradient distortions and wrap-around artefacts, due to the distinct borders and central location of the ventricles [6]. This may partly explain why LVV was a significant predictor of PIRA and d-SPMS in this real-world study of clinical-quality MRI scans, while WBV and GMV were not. Furthermore, the pathological processes that drive periventricular atrophy may occur relatively early in the MS disease course [37]. Thus, in PwMS with a severe disease trajectory, the associated lateral ventricular enlargement may become apparent at an earlier stage than other cross-sectional MRI markers of neuroaxonal loss.

Our study also provides insights into the association between cross-sectional LVV and PIRA. Cagol et al. [7] found that ventricular enlargement occurs more rapidly in patients with PIRA compared to propensity score-matched stable patients; our cross-sectional result aligns with their longitudinal finding. Our study also produced novel findings regarding cross-sectional volumetric measures and sustained CDI. Higher WBV, GMV and WMV and lower LVV were each associated with an increased hazard of achieving CDI, suggesting that the degree of neuroaxonal loss at baseline is a key determinant of the capacity for disability reversal.

There was only one significant association between a lesion volume measure and disability outcome in this study, which was the association between higher BHLV and subsequent conversion to d-SPMS. This aligns with previous findings that BHLV correlates more strongly than T2LV with clinical outcomes in both cross-sectional and longitudinal studies [5, 24–26]. Persistent black holes represent severe demyelination, axonal loss and matrix destruction, whereas T2 hyperintensities represent increased water content in the tissue, which is less specific for irreversible tissue loss [1, 2].

Our findings support the concept that both diffuse and, to a lesser extent, focal tissue loss contribute to disability progression in RRMS. However, the associations between cross-sectional brain volumetric measures and the hazard of subsequent disability progression events were modest, even when statistically significant. This highlights the importance of other prognostic factors in RRMS, which include age, comorbidities such as vascular disease and psychiatric conditions, lifestyle factors such as smoking and exercise, spinal cord lesions and use of disease-modifying therapy [3, 38, 39]. The implementation of cross-sectional brain volumetric measures in clinical practice would enable clinicians to identify PwMS who may be at a higher risk of disability progression due to having relatively low brain volumes. Particular focus could then be placed on early optimisation of modifiable risk factors in these cases.

HET, encompassing cladribine, natalizumab, anti-CD20 monoclonal antibodies, alemtuzumab and AHSCT, was associated with a 69% reduction in relapse rate. Early initiation of specific HETs as first-line treatments in MS has been shown to improve the likelihood of attaining CDI [40] and reduce the risk of CDW [41]. Less advanced brain atrophy, coupled with greater neural reserve early in the disease course, may contribute to this treatment response.

### Limitations

There are a number of limitations associated with our study. First, registry data may be more susceptible to quality issues. However, our use of data from the MSBase Registry also has many advantages. We were able to recruit a very large international cohort with data derived from clinically acquired MRI, which maximises the generalisability of our results. Another limitation relates to the cross-sectional measurement of BHLV: most acute T1-hypointense lesions become isointense over a period of months as oedema resolves and remyelination occurs [2]. It was not possible to separate acute from persistent black holes in this study. A lack of contrast-enhanced images for most participants precluded radiological identification of clinically silent acute inflammation. Brain volume, particularly WMV [42], can increase with acute inflammation and markedly decrease following treatment, most notably with corticosteroids, but also with DMTs [8, 19]. To mitigate this effect, we excluded scans performed within 2 months of pulsed corticosteroids. We also conducted a sensitivity analysis excluding 110 participants who had either started their first DMT within the 6 months preceding the MRI, or had initiated a DMT within 6 months of the MRI following a treatment gap of at least 6 months. The results of this sensitivity analysis were largely consistent with the primary analysis, supporting the robustness of the findings.

Lastly, the icobrain ms software used in this study could not provide volumetric measurements of individual cerebral grey matter regions, such as the thalami, and spinal cord volumetric measurements were also unavailable. Cervical spinal cord area and volume have been shown to correlate with disability in MS [43], and some upper cervical cord measurement techniques can be applied to brain MRI scans [44]. Atrophy of specific brain structures, such as the thalami, may also serve as important prognostic markers in MS [45]. Future large observational studies should include regional brain and spinal cord volumetric measures to assess their independent associations with disability outcomes.

## Conclusion

This study provides additional, real-world evidence that cross-sectional MRI measures of brain volume loss are relevant to disability outcomes in RRMS. Our findings suggest that LVV is a particularly informative prognostic marker, as it was significantly associated with all sustained disability progression and improvement outcomes. Cross-sectional volumetric measures face fewer technical barriers to clinical implementation than longitudinal measures, and could provide immediate prognostic information that helps to facilitate tailored and timely treatments for PwMS. Our findings support efforts to integrate these measures into standard clinical practice.

## Supplementary Information

Below is the link to the electronic supplementary material.

Supplementary file1 (PDF 2185 KB)

## Data Availability

MSBase is a data processor, and warehouses data from individual principal investigators who agree to share their datasets on a project-by-project basis. Each principal investigator will need to be approached individually for permission to access the datasets.
