# Supplementary material for: Cross-sectional brain volumetric measures and disability outcomes in relapsing–remitting multiple sclerosis
Source: J Neurol. 2026 Apr 1;273(4):243. doi: 10.1007/s00415-026-13788-z (PMC13043540; doi:10.1007/s00415-026-13788-z)
Supplement: Supplementary file 1 — Supplementary file1 (PDF 2185 KB) [file 415_2026_13788_MOESM1_ESM.pdf]

Supplementary Material

Cross-sectional brain volumetric measures and disability outcomes in relapsing-remitting multiple sclerosis

Contents

icobrain ms MRI analysis methodology **3**

icobrain ms 3.1 3

icobrain ms 5.13 3

Supplementary Table 1. Summary of disease-modifying therapies received by participants during follow-up and treatment thresholds used to calculate treatment intervals **4**

Supplementary Table 2. List of MRI scanners used **5**

Supplementary Table 3. Comparison of clinical and MRI characteristics between participants with and without CDW **6**

Supplementary Table 4. Comparison of clinical and MRI characteristics between participants with and without PIRA **7**

Supplementary Table 5. Comparison of clinical and MRI characteristics between participants with and without d-SPMS **8**

Supplementary Table 6. Comparison of clinical and MRI characteristics between participants with and without CDI **9**

Supplementary Table 7. Multivariable Cox regression analyses of associations between brain volumetric measures and disability outcomes featuring one MRI metric per model ... **10**

Supplementary Table 8. Multivariable Cox regression sensitivity analysis 1 .... **11**

Supplementary Table 9. Multivariable Cox regression sensitivity analysis 2 .... **12**

Supplementary Figure 1. Comparison of brain MRI volumetric measures between the cohorts analysed with icobrain ms 3.1 and icobrain ms 5.13 .... **13**

Supplementary Figure 2. Survival curves for time to CDW for baseline WBV, GMV, WMV, LVV, T2LV and BHLV **14**

Supplementary Figure 3. Survival curves for time to d-SPMS for baseline WBV, GMV, WMV, LVV, T2LV and BHLV **15**

Supplementary Figure 4. Survival curves for time to PIRA for baseline WBV, GMV, T2LV and BHLV **16**

Supplementary Figure 5. Survival curves for time to CDI for baseline GMV, WMV, T2LV and BHLV **17**

Supplementary References **17**

icobrain ms MRI analysis methodology

icobrain ms 3.1

In the icobrain ms 3.1 [1] cross-sectional analysis pipeline, the input T2-weighted fluid attenuated inversion recovery (FLAIR) image is rigidly co-registered with the input 3D T1-weighted image, and the T1-weighted image is skull-stripped. A probabilistic model is applied to segment the skull-stripped T1-weighted image using probabilistic tissue priors. The model assumes Gaussian-distributed tissue intensities, a smoothly varying bias field for intensity non-uniformities and contains a spatial consistency model based on a Markov Random Field. Optimised through an expectation-maximisation algorithm, the process iteratively refines tissue class parameters and bias field estimates until convergence, resulting in bias-corrected segmentation into grey matter, white matter, and cerebrospinal fluid (CSF). Using these three tissue class segmentations as prior information, lesions are then segmented from the co-registered T2 FLAIR image, followed by lesion filling on the T1-weighted image. T2 FLAIR lesions are identified as hyperintense regions consisting of at least five adjacent voxels with intensities exceeding the mean grey matter intensity by more than two standard deviations on the T2 FLAIR image. Black holes are identified as parts of T2 FLAIR lesions with T1 intensities at least three standard deviations below the mean white matter T1 intensity. The T1-weighted image is segmented again after lesion filling, resulting in the final volume measures of grey matter, white matter and CSF.

icobrain ms 5.13

The icobrain ms 5.13 [2, 3] cross-sectional analysis pipeline utilises a deep learning model to process the 3D T1-weighted image via a single-input, dual-output 3D convolutional neural network (CNN) to produce estimated multi-label masks for brain tissues (background, white matter, grey matter and CSF) and brain structures (background plus 22 brain structures, including the lateral ventricles). Similarly, a 3D CNN is employed to process the T2 FLAIR image to segment white matter lesions and classify them based on location (periventricular, juxtacortical, deep white matter or infratentorial). The models were trained on comprehensive datasets of pre- and post-contrast 3D T1-weighted images and 2D/3D T2 FLAIR images respectively, with ground truth annotations derived from predictions made by the earlier icobrain version (icobrain 5.9) along with minor manual corrections where necessary.

Supplementary Table 1. Summary of disease-modifying therapies received by participants during follow-up and treatment thresholds used to calculate treatment intervals

DMT	Number of participants treated (n = 1598)	Time covered by DMT after last dose (if no new DMT started sooner), days
No DMT during follow-up	34	NA
Natalizumab	663	70
Fingolimod	653	14
Ocrelizumab	483	365
Interferon beta-1a	228	14
Dimethyl fumarate	225	14
Cladribine	166	Indefinite/until next DMT
Glatiramer acetate	141	14
Alemtuzumab	104	Indefinite/until next DMT
Teriflunomide	104	14
Ofatumumab	64	84
Interferon beta-1b	58	14
Rituximab	40	365
ASCT	21	Indefinite/until next DMT
Azathioprine	21	14
IVIg	18	70
Cyclophosphamide	14	90
Methotrexate	8	14
Siponimod	6	14
Diroximel fumarate	5	14
Mycophenolate	4	14
Daclizumab	4	42
Ponesimod	3	14
Ozanimod	2	14

Abbreviations: DMT: disease-modifying therapy; NA: not applicable.

Supplementary Table 2. List of MRI scanners used

Vendor	Model	Field Strength	Site[a]
GE	Discovery MR750	3.0T	1
GE	Genesis Signa	1.5T	2
GE	Optima MR450w	1.5T	6
GE	Signa Explorer	1.5T	5
GE	Signa HDxt	1.5T, 3.0T	1, 6
Philips	Achieva	3.0T	1, 6, 8
Philips	Achieva dStream	3.0T	8
Philips	Gyroscan NT	1.5T	9
Philips	Ingenia	1.5, 3.0T	1, 4, 6
Philips	Intera	1.5T	9
Siemens	Aera	1.5T	1, 2, 3, 4, 7
Siemens	Avanto	1.5T	1, 7
Siemens	Avanto Fit	1.5T	7
Siemens	Prisma	3.0T	3
Siemens	Prisma Fit	3.0T	2, 7
Siemens	Skyra	3.0T	1, 2, 5, 7, 9
Siemens	Skyra Fit	3.0T	4, 6
Siemens	Symphony Tim	1.5T	7
Siemens	Trio Tim	3.0T	2, 7
Siemens	Verio	3.0T	4, 6

[a.] Site 1: Brain and Mind Centre, Sydney, Australia.
Site 2: Royal Melbourne Hospital, Melbourne, Australia.
Site 3: John Hunter Hospital, Newcastle, Australia.
Site 4: Box Hill Hospital, Melbourne, Australia.
Site 5: The Alfred Hospital, Melbourne, Australia.
Site 6: Cliniques Universitaires Saint-Luc, Brussels, Belgium.
Site 7: Ghent University Hospital, Ghent, Belgium.
Site 8: University Hospital "G. Rodolico-San Marco", Catania, Italy.
Site 9: General University Hospital, Prague, Czech Republic.

Supplementary Table 3. Comparison of clinical and MRI characteristics between participants with and without CDW

**Characteristic**	**CDW**<br>**n = 371 (23.2%)**	**No CDW**<br>**n = 1227 (76.8%)**	**Standardised difference**	***p* value**
Age at baseline (years), mean (SD)	42.2 (11.0)	39.6 (10.5)	0.25	< 0.001[a]
Female, n (%)	277 (74.7)	913 (74.4)	0.01	0.98[b]
Disease duration at baseline (years), median (IQR)	9.4 (5.3–14.7)	7.1 (3.34–13.0)	0.26	< 0.001[c]
Number of relapses within 2 years of baseline, median (IQR)	0 (0–2)	0 (0–1)	0.16	0.02[c]
CIS at baseline, n (%)	11 (3.0)	36 (2.9)	< 0.01	1[b]
Time from first DMT initiation to baseline (years), median (IQR)	5.9 (3.0–9.6)	4.9 (2.1–8.8)	0.16	0.002[c]
No DMT use before baseline, n (%)	27 (7.3)	133 (10.8)	0.12	0.06[b]
Taking any DMT at baseline, n (%)	334 (90.0)	1037 (84.5)	0.16	0.01[b]
Taking HET at baseline, n (%)	120 (32.4)	413 (33.7)	0.03	0.68[b]
Took ≥1 HET during follow-up, n (%)	271 (73.1)	825 (67.2)	0.13	0.04[b]
EDSS score at baseline, median (IQR)	2 (1–3.5)	2 (1–3)	0.17	0.004[c]
EDSS score at final follow-up, median (IQR)	4 (2.5–6)	2 (1–3)	1.34	< 0.001[c]
Duration of follow-up (years), median (IQR)	8.8 (6.8–10.2)	7.4 (4.5–9.4)	0.49	< 0.001[c]
WBV (mL), mean (SD)	1514.6 (69.7)	1540.5 (71.1)	0.36	< 0.001[a]
GMV (mL), mean (SD)	897.3 (50.2)	914.1 (54.1)	0.32	< 0.001[a]
WMV (mL), mean (SD)	617.3 (44.1)	626.4 (41.5)	0.22	< 0.001[a]
LVV (mL), median (IQR)	30.0 (23.0–43.0)	27.0 (19.0–37.0)	0.30	< 0.001[c]
T2LV (mL), median (IQR)	4.8 (2.3–9.4)	3.5 (1.4–7.9)	0.27	< 0.001[c]
BHLV (mL), median (IQR)	2.7 (1.2–5.5)	2.0 (0.8–4.9)	0.22	< 0.001[c]

Abbreviations: BHLV: black hole lesion volume; CDW: confirmed disability worsening; CIS: clinically isolated syndrome; DMT: disease-modifying therapy; EDSS: Expanded Disability Status Scale; GMV: normalised grey matter volume; HET: high-efficacy disease-modifying therapy; LVV: normalised lateral ventricular volume; T2LV: T2-weighted fluid attenuated inversion recovery lesion volume; WBV: normalised whole brain volume; WMV: normalised white matter volume.

[a] Independent samples t-test.

[b] χ^2 test.

[c] Mann-Whitney U test.

Supplementary Table 4. Comparison of clinical and MRI characteristics between participants with and without PIRA

Characteristic	PIRA<br>n = 248 (15.5%)	No PIRA<br>n = 1350 (84.5%)	Standardised difference	*p* value
Age at baseline (years), mean (SD)	44.5 (11.3)	39.4 (10.4)	0.48	< 0.001[a]
Female, n (%)	178 (71.8)	1012 (75.0)	0.07	0.33[b]
Disease duration at baseline (years), median (IQR)	10.0 (5.6–15.1)	7.2 (3.5–13.0)	0.29	< 0.001[c]
Number of relapses within 2 years of baseline, median (IQR)	0 (0–1)	0 (0–1)	0.09	0.25[c]
CIS at baseline, n (%)	5 (2.0)	42 (3.1)	0.07	0.46[b]
Time from first DMT initiation to baseline (years), median (IQR)	6.0 (3.1–10.4)	5.0 (2.1–8.9)	0.21	0.002[c]
No DMT use before baseline, n (%)	18 (7.3)	142 (10.5)	0.11	0.15[b]
Taking any DMT at baseline, n (%)	220 (88.7)	1151 (85.3)	0.10	0.18[b]
Taking HET at baseline, n (%)	91 (36.7)	442 (32.7)	0.09	0.25[b]
Took ≥1 HET during follow-up, n (%)	172 (69.4)	924 (68.4)	0.02	0.83[b]
EDSS score at baseline, median (IQR)	2 (1–4)	2 (1–3)	0.25	0.002[c]
EDSS score at final follow-up, median (IQR)	4.5 (2.5–6)	2 (1–3)	1.35	< 0.001[c]
Duration of follow-up (years), median (IQR)	8.2 (6.3–9.7)	7.7 (4.7–9.6)	0.24	0.002[c]
WBV (mL), mean (SD)	1510.9 (72.3)	1538.8 (70.6)	0.39	< 0.001[a]
GMV (mL), mean (SD)	896.1 (51.4)	912.8 (53.7)	0.31	< 0.001[a]
WMV (mL), mean (SD)	614.8 (46.7)	626.0 (41.2)	0.27	< 0.001[a]
LVV (mL), median (IQR)	32.0 (25.0–45.3)	27.0 (19.0–38.0)	0.38	< 0.001[c]
T2LV (mL), median (IQR)	5.0 (2.4–9.7)	3.6 (1.5–7.9)	0.31	< 0.001[c]
BHLV (mL), median (IQR)	2.9 (1.4–6.5)	2.1 (0.8–4.8)	0.31	< 0.001[c]

Abbreviations: BHLV: black hole lesion volume; CIS: clinically isolated syndrome; DMT: disease-modifying therapy; EDSS: Expanded Disability Status Scale; GMV: normalised grey matter volume; HET: high-efficacy disease-modifying therapy; LVV: normalised lateral ventricular volume; PIRA: progression independent of relapse activity; T2LV: T2-weighted fluid attenuated inversion recovery lesion volume; WBV: normalised whole brain volume; WMV: normalised white matter volume.

[a] Independent samples t-test.

[b] χ^2 test.

[c] Mann-Whitney U test.

Supplementary Table 5. Comparison of clinical and MRI characteristics between participants with and without d-SPMS

**Characteristic**	**d-SPMS**<br>**n = 169 (10.6%)**	**No d-SPMS**<br>**n = 1429 (89.4%)**	**Standardised difference**	***p* value**
Age at baseline (years), mean (SD)	45.1 (11.0)	39.6 (10.5)	0.52	< 0.001[a]
Female, n (%)	120 (71.0)	1070 (74.9)	0.09	0.32[b]
Disease duration at baseline (years), median (IQR)	11.3 (7.1–17.0)	7.2 (3.6–12.9)	0.53	< 0.001[c]
Number of relapses within 2 years of baseline, median (IQR)	1 (0–2)	0 (0–1)	0.17	0.04[c]
CIS at baseline, n (%)	3 (1.8)	44 (3.1)	0.08	0.47[d]
Time from first DMT initiation to baseline (years), median (IQR)	7.2 (3.8–11.2)	5.0 (2.2–8.7)	0.38	< 0.001[c]
No DMT use before baseline, n (%)	10 (5.9)	150 (10.5)	0.15	0.08[b]
Taking any DMT at baseline, n (%)	154 (91.1)	1217 (85.2)	0.17	0.05 [b]
Taking HET at baseline, n (%)	68 (40.2)	465 (32.5)	0.16	0.06 [b]
Took ≥1 HET during follow-up, n (%)	134 (79.3)	962 (67.3)	0.26	0.002[b]
EDSS score at baseline, median (IQR)	3.5 (2.5–4.5)	2 (1–2.5)	1.17	< 0.001 [c]
EDSS score at final follow-up, median (IQR)	6 (5–6.5)	2 (1–3)	2.29	< 0.001 [c]
Duration of follow-up (years), median (IQR)	8.5 (6.8–10.1)	7.7 (4.7–9.5)	0.43	< 0.001 [c]
WBV (mL), mean (SD)	1497.3 (71.5)	1538.9 (70.3)	0.59	< 0.001 [a]
GMV (mL), mean (SD)	887.6 (48.4)	912.9 (53.6)	0.48	< 0.001 [a]
WMV (mL), mean (SD)	609.8 (47.6)	626.0 (41.3)	0.39	< 0.001[a]
LVV (mL), median (IQR)	36.0 (27.0–52.0)	27.0 (19.0–37.0)	0.62	< 0.001 [c]
T2LV (mL), median (IQR)	6.2 (2.9–13.8)	3.6 (1.5–7.6)	0.60	< 0.001 [c]
BHLV (mL), median (IQR)	4.0 (1.8–8.5)	2.0 (0.8–4.6)	0.58	< 0.001 [c]

Abbreviations: BHLV: black hole lesion volume; CIS: clinically isolated syndrome; DMT: disease-modifying therapy; EDSS: Expanded Disability Status Scale; GMV: normalised grey matter volume; HET: high-efficacy disease-modifying therapy; LVV: normalised lateral ventricular volume; d-SPMS: data-driven secondary progressive multiple sclerosis; T2LV: T2-weighted fluid attenuated inversion recovery lesion volume; WBV: normalised whole brain volume; WMV: normalised white matter volume.

[a] Independent samples t-test.

[b] χ^2 test.

[c] Mann-Whitney U test.

[d] Fisher's exact test.

Supplementary Table 6. Comparison of clinical and MRI characteristics between participants with and without CDI

**Characteristic**	**CDI**<br>**n = 107 (12.0%)**	**No CDI**<br>**n = 784 (88.0%)**	**Standardised difference**	***p* value**
Age at baseline (years), mean (SD)	38.9 (10.0)	42.9 (10.9)	0.38	< 0.001[a]
Female, n (%)	84 (78.5)	565 (72.1)	0.14	0.20[b]
Disease duration at baseline (years), median (IQR)	5.8 (1.9–13.4)	10.3 (5.6–15.9)	0.42	< 0.001[c]
Number of relapses within 2 years of baseline, median (IQR)	1 (0–2)	1 (0–2)	0.01	0.80[c]
CIS at baseline, n (%)	6 (5.6)	10 (1.3)	0.32	0.008[d]
Time from first DMT initiation to baseline (years), median (IQR)	4.8 (1.7–7.6)	6.6 (3.0–10.8)	0.33	0.002[c]
No DMT use before baseline, n (%)	27 (25.2)	50 (6.4)	0.67	< 0.001[b]
Taking any DMT at baseline, n (%)	74 (69.2)	704 (89.8)	0.62	< 0.001[b]
Taking HET at baseline, n (%)	25 (30.5)	302 (38.5)	0.17	0.003[b]
Took ≥1 HET during follow-up, n (%)	80 (74.8)	561 (71.6)	0.07	0.56[b]
EDSS score at baseline, median (IQR)	3 (2–4)	3 (2–4)	0.04	0.68[c]
EDSS score at final follow-up, median (IQR)	1 (1.5–2)	3.5 (2.5–5)	1.27	< 0.001[c]
Duration of follow-up (years), median (IQR)	8.1 (5.4–9.7)	8.0 (5.1–9.8)	0.01	0.96[c]
WBV (mL), mean (SD)	1559.8 (67.5)	1510.6 (72.0)	0.69	< 0.001[a]
GMV (mL), mean (SD)	923.8 (53.0)	893.5 (51.0)	0.59	< 0.001[a]
WMV (mL), mean (SD)	636.0 (38.5)	617.1 (44.9)	0.43	< 0.001[a]
LVV (mL), median (IQR)	25.0 (18.0–38.0)	32.0 (23.0–45.0)	0.35	< 0.001[c]
T2LV (mL), median (IQR)	3.4 (1.1–7.7)	5.3 (2.4–10.4)	0.33	< 0.001[c]
BHLV (mL), median (IQR)	2.1 (0.6–5.3)	2.9 (1.3–6.5)	0.2	0.003[c]

Abbreviations: BHLV: black hole lesion volume; CDI: confirmed disability improvement; CIS: clinically isolated syndrome; DMT: disease-modifying therapy; EDSS: Expanded Disability Status Scale; GMV: normalised grey matter volume; HET: high-efficacy disease-modifying therapy; LVV: normalised lateral ventricular volume; T2LV: T2-weighted fluid attenuated inversion recovery lesion volume; WBV: normalised whole brain volume; WMV: normalised white matter volume.

[a] Independent samples t-test.

[b] χ^2 test.

[c] Mann-Whitney U test.

[d] Fisher's exact test.

Supplementary Table 7. Multivariable Cox regression analyses of associations between brain volumetric measures and disability outcomes featuring one MRI metric per model

**MRI predictor**	**CDW**<br>**HR (95% CI)**	**PIRA**<br>**HR (95% CI)**	**d-SPMS**<br>**HR (95% CI)**	**CDI**<br>**HR (95% CI)**
WBV, mL[a]	0.996<br>(0.995–0.998),<br>***p* < 0.001**	0.998<br>(0.996–0.9996),<br>***p* = 0.02**	0.997<br>(0.995–0.9997),<br>***p* = 0.03**	1.008<br>(1.005–1.011),<br>***p* < 0.001**
GMV, mL[a]	0.997<br>(0.994–0.999),<br>***p* = 0.008**	1.000<br>(0.997–1.003),<br>*p* = 0.9	0.999<br>(0.996–1.003),<br>*p* = 0.7	1.009<br>(1.005–1.014),<br>***p* < 0.001**
WMV, mL[a]	0.995<br>(0.993–0.998),<br>***P* < 0.001**	0.995<br>(0.992–0.998),<br>***p* = 0.003**	0.995<br>(0.991–0.999)<br>***p* = 0.01**	1.008<br>(1.003–1.012)<br>***p* = 0.001**
LVV, mL[a]	1.009<br>(1.005–1.013),<br>***p* < 0.001**	1.009<br>(1.004–1.013),<br>***p* < 0.001**	1.010<br>(1.005–1.016),<br>***p* < 0.001**	0.986<br>(0.975–0.998),<br>***p* = 0.02**
BHLV, mL[a]	1.028<br>(1.005–1.052),<br>***p* = 0.02**	1.033<br>(1.006–1.061),<br>***p* = 0.02**	1.039<br>(1.011–1.068),<br>***p* = 0.007**	0.974<br>(0.928–1.022),<br>*p* = 0.3
T2LV, mL[a]	1.024<br>(1.007–1.041),<br>***p* = 0.005**	1.025<br>(1.005–1.045),<br>***p* = 0.01**	1.029<br>(1.007–1.051),<br>***p* = 0.008**	0.962<br>(0.928–0.998),<br>***p* = 0.04**

[a] Covariates in all models were age, disease duration and EDSS at baseline, number of relapses in the two years preceding baseline, proportion of time on DMT pre-baseline, sex, icobrain ms software version and DMT as a time-varying covariate.

Bold values indicate statistically significant results.

Abbreviations: BHLV: black hole lesion volume; CDI: confirmed disability improvement; CDW: confirmed disability worsening; GMV: normalised grey matter volume; HR: hazard ratio; LVV: normalised lateral ventricular volume; MRI: magnetic resonance imaging; PIRA: progression independent of relapse activity; d-SPMS: data-driven secondary progressive multiple sclerosis; T2LV: T2-weighted fluid attenuated inversion recovery lesion volume; WBV: normalised whole brain volume; WMV: normalised white matter volume.

Supplementary Table 8. Multivariable Cox regression sensitivity analyses of associations between brain volumetric measures and disability outcomes stratified by study site

MRI predictor	CDW<br>HR (95% CI)	PIRA<br>HR (95% CI)	d-SPMS<br>HR (95% CI)	CDI<br>HR (95% CI)
WBV, mL[a]	0.998<br>(0.996–0.999),<br>***p* = 0.01**	0.999<br>(0.997–1.001),<br>*p* = 0.4	0.999<br>(0.997–1.002),<br>*p* = 0.7	1.007<br>(1.003–1.011),<br>***p* < 0.001**
GMV, mL[a]	0.998<br>(0.996–1.001),<br>*p* = 0.2	1.001<br>(0.998–1.004),<br>*p* = 0.6	1.001<br>(0.996–1.005),<br>*p* = 0.8	1.007<br>(1.002–1.012),<br>***p* = 0.009**
WMV, mL[a]	0.997<br>(0.994–0.999),<br>***p* = 0.01**	0.997<br>(0.993–1.000),<br>*p* = 0.08	0.998<br>(0.994–1.003)<br>*p* = 0.4	1.008<br>(1.003–1.012)<br>***p* = 0.002**
LVV, mL[a]	1.007<br>(1.002–1.011),<br>***p* = 0.002**	1.006<br>(1.001–1.011),<br>***p* = 0.02**	1.007<br>(1.001–1.014),<br>***p* = 0.02**	0.987<br>(0.975–0.9995),<br>***p* = 0.04**
BHLV, mL[b]	1.018<br>(0.993–1.044),<br>*p* = 0.2	1.024<br>(0.994–1.054),<br>*p* = 0.1	1.033<br>(1.003–1.064),<br>***p* = 0.03**	1.012<br>(0.965–1.060),<br>*p* = 0.6
T2LV, mL[b]	1.013<br>(0.995–1.031),<br>*p* = 0.2	1.020<br>(0.998–1.042),<br>*p* = 0.07	1.027<br>(1.004–1.051),<br>***p* = 0.02**	1.000<br>(0.966–1.036),<br>*p* = 0.996

[a] BHLV was included as a covariate alongside age, disease duration and EDSS at baseline, number of relapses in the two years preceding baseline, proportion of time on DMT pre-baseline, sex, icobrain ms software version and DMT as a time-varying covariate.

[b] WBV was included as a covariate alongside age, disease duration and EDSS at baseline, number of relapses in the two years preceding baseline, proportion of time on DMT pre-baseline, sex, icobrain ms software version and DMT as a time-varying covariate.

Bold values indicate statistically significant results.

Abbreviations: BHLV: black hole lesion volume; CDI: confirmed disability improvement; CDW: confirmed disability worsening; GMV: normalised grey matter volume; HR: hazard ratio; LVV: normalised lateral ventricular volume; MRI: magnetic resonance imaging; PIRA: progression independent of relapse activity; d-SPMS: data-driven secondary progressive multiple sclerosis; T2LV: T2-weighted fluid attenuated inversion recovery lesion volume; WBV: normalised whole brain volume; WMV: normalised white matter volume.

Supplementary Table 9. Multivariable Cox regression sensitivity analyses of associations between brain volumetric measures and disability outcomes after excluding participants initiating DMT within six months of MRI.

MRI predictor	CDW<br>n = 359 of 1488<br>HR (95% CI)	PIRA<br>n = 241 of 1488<br>HR (95% CI)	d-SPMS<br>n = 163 of 1488<br>HR (95% CI)	CDI<br>n = 94 of 838<br>HR (95% CI)
WBV, mL[a]	0.997<br>(0.995–0.998),<br>$\boldsymbol{p}$ **< 0.001**	0.999<br>(0.996–1.001),<br>$p = 0.2$	0.999<br>(0.996–1.001),<br>$p = 0.3$	1.007<br>(1.004–1.011),<br>$\boldsymbol{p}$ **< 0.001**
GMV, mL[a]	0.997<br>(0.994–0.9996),<br>$\boldsymbol{p}$ **= 0.02**	1.001<br>(0.998–1.004),<br>$p = 0.6$	1.000<br>(0.996–1.004),<br>$p = 0.9$	1.008<br>(1.003–1.013),<br>$\boldsymbol{p}$ **= 0.002**
WMV, mL[a]	0.996<br>(0.993–0.999),<br>$\boldsymbol{p}$ **= 0.004**	0.996<br>(0.993–0.995),<br>$\boldsymbol{p}$ **= 0.03**	0.997<br>(0.993–1.001)<br>$p = 0.1$	1.006<br>(1.001–1.012)<br>$\boldsymbol{p}$ **= 0.01**
LVV, ml[a]	1.008<br>(1.003–1.013),<br>$\boldsymbol{p}$ **< 0.001**	1.007<br>(1.001–1.012),<br>$\boldsymbol{p}$ **= 0.02**	1.009<br>(1.002–1.015),<br>$\boldsymbol{p}$ **= 0.01**	0.989<br>(0.976–1.003),<br>$p = 0.1$
BHLV, ml[b]	1.013<br>(0.987–1.039),<br>$p = 0.4$	1.026<br>(0.997–1.057),<br>$p = 0.08$	1.033<br>(1.002–1.065),<br>$\boldsymbol{p}$ **= 0.04**	1.006<br>(0.961–1.053),<br>$p = 0.8$
T2LV, ml[b]	1.011<br>(0.993–1.030),<br>$p = 0.2$	1.019<br>(0.997–1.042),<br>$p = 0.09$	1.024<br>(1.000–1.049),<br>$p = 0.05$	0.987<br>(0.952–1.023),<br>$p = 0.5$

[a] BHLV was included as a covariate alongside age, disease duration and EDSS at baseline, number of relapses in the two years preceding baseline, proportion of time on DMT pre-baseline, sex, icobrain ms software version and DMT as a time-varying covariate.

[b] WBV was included as a covariate alongside age, disease duration and EDSS at baseline, number of relapses in the two years preceding baseline, proportion of time on DMT pre-baseline, sex, icobrain ms software version and DMT as a time-varying covariate.

Participants were excluded if they had commenced their first DMT or restarted DMT after a treatment-free period of six months or more within the six months preceding the MRI scan.

Bold values indicate statistically significant results.

Abbreviations: BHLV: black hole lesion volume; CDI: confirmed disability improvement; CDW: confirmed disability worsening; GMV: normalised grey matter volume; HR: hazard ratio; LVV: normalised lateral ventricular volume; MRI: magnetic resonance imaging; PIRA: progression independent of relapse activity; d-SPMS: data-driven secondary progressive multiple sclerosis; T2LV: T2-weighted fluid attenuated inversion recovery lesion volume; WBV: normalised whole brain volume; WMV: normalised white matter volume.

Supplementary Figure 1. Comparison of brain MRI volumetric measures between the cohorts analysed with icobrain ms 3.1 (eight centres, n = 1402) and icobrain ms 5.13 (one centre: The Alfred Hospital, n = 196)

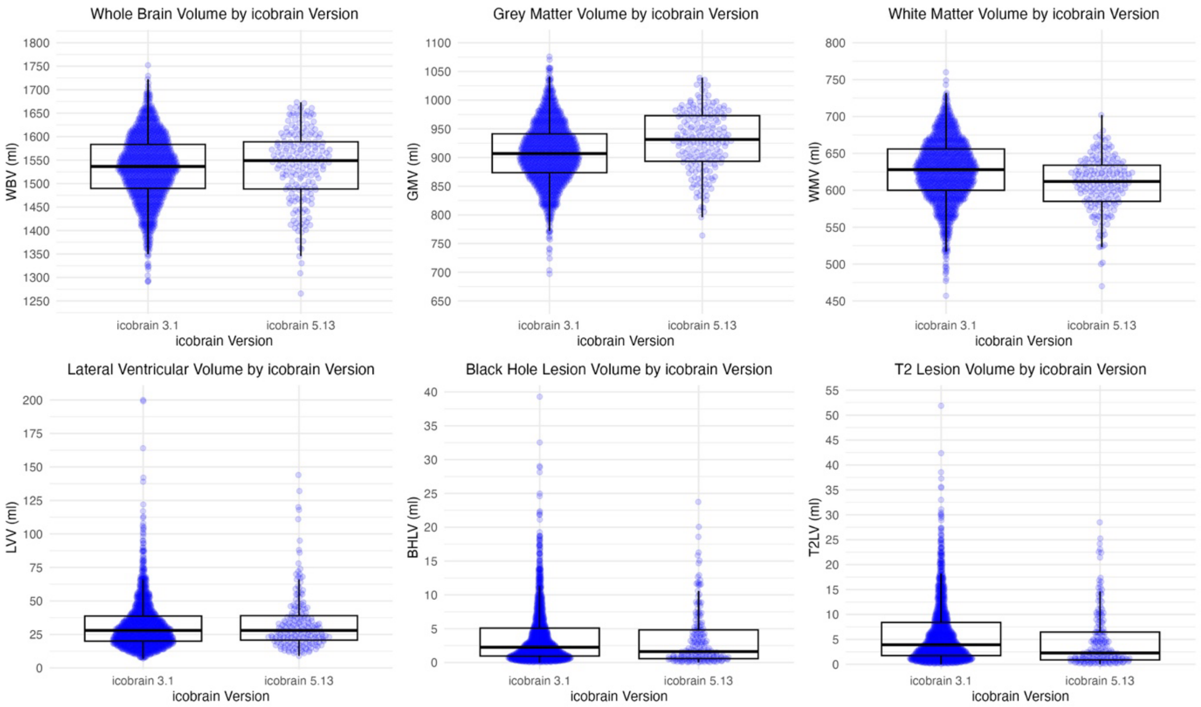

Abbreviations: BHLV: black hole lesion volume; GMV: normalised grey matter volume; LVV: normalised lateral ventricular volume; T2LV: T2 lesion volume; WBV: normalised whole brain volume; WMV: normalised white matter volume.

Supplementary Figure 2. Survival curves for time to CDW for baseline WBV (A), GMV (B), WMV (C), LVV (D), T2LV (E) and BHLV (F)

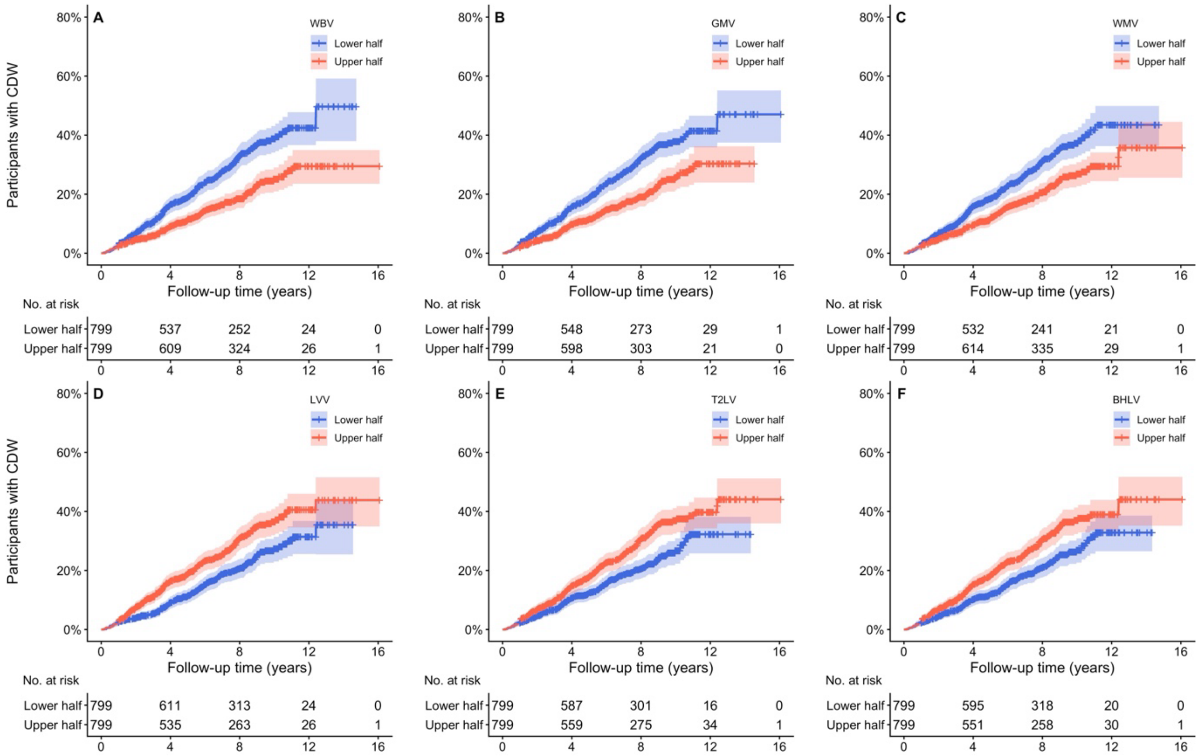

The red curve represents the upper half and the blue curve represents the lower half of the cohort (median split was performed) for the associated brain MRI volumetric measurement, with the lighter shade reflecting the 95% CI. Abbreviations: BHLV: black hole lesion volume; CDW: confirmed disability worsening; GMV: normalised grey matter volume; LVV: normalised lateral ventricular volume; T2LV: T2 lesion volume; WBV: normalised whole brain volume; WMV: normalised white matter volume.

Supplementary Figure 3. Survival curves for time to d-SPMS for baseline WBV (A), GMV (B), WMV (C), LVV (D), T2LV (E) and BHLV (F)

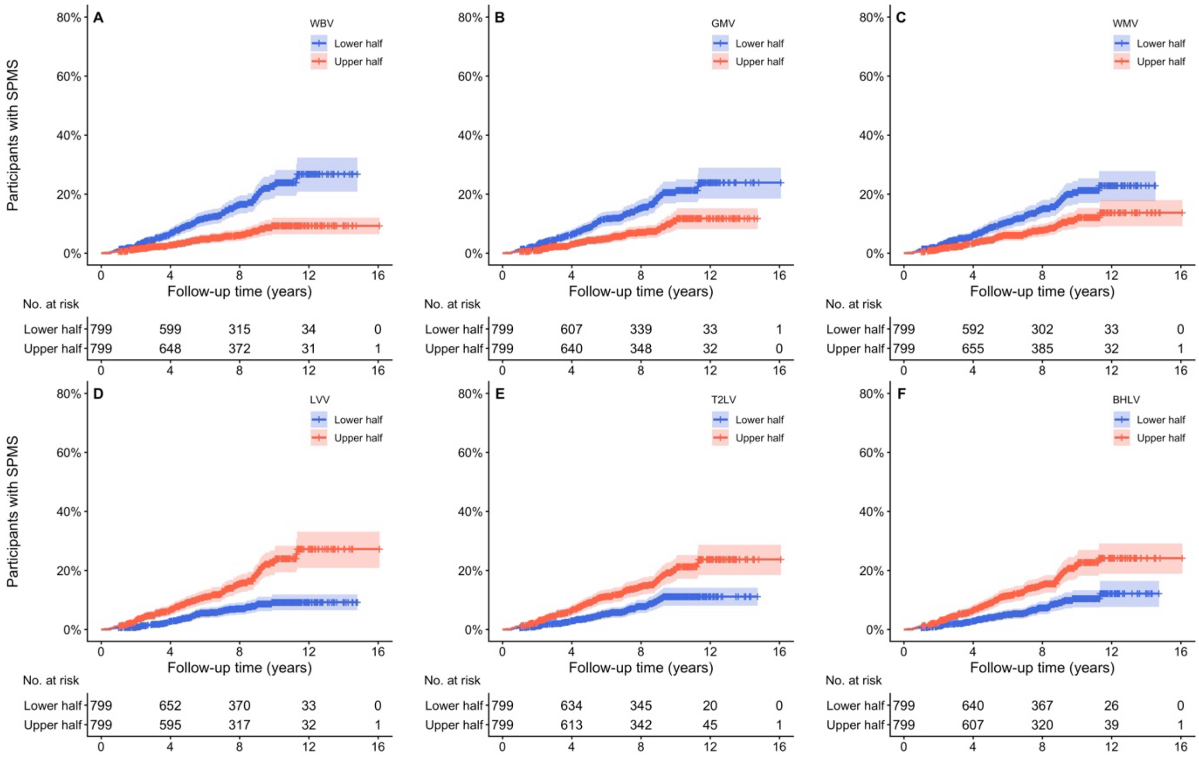

The red curve represents the upper half and the blue curve represents the lower half of the cohort (median split was performed) for the associated brain MRI volumetric measurement, with the lighter shade reflecting the 95% CI. Abbreviations: BHLV: black hole lesion volume; GMV: normalised grey matter volume; LVV: normalised lateral ventricular volume; d-SPMS: data-driven secondary progressive MS; T2LV: T2 lesion volume; WBV: normalised whole brain volume; WMV: normalised white matter volume.

Supplementary Figure 4. Survival curves for time to PIRA for baseline WBV (A), GMV (B), T2LV (C) and BHLV (D)

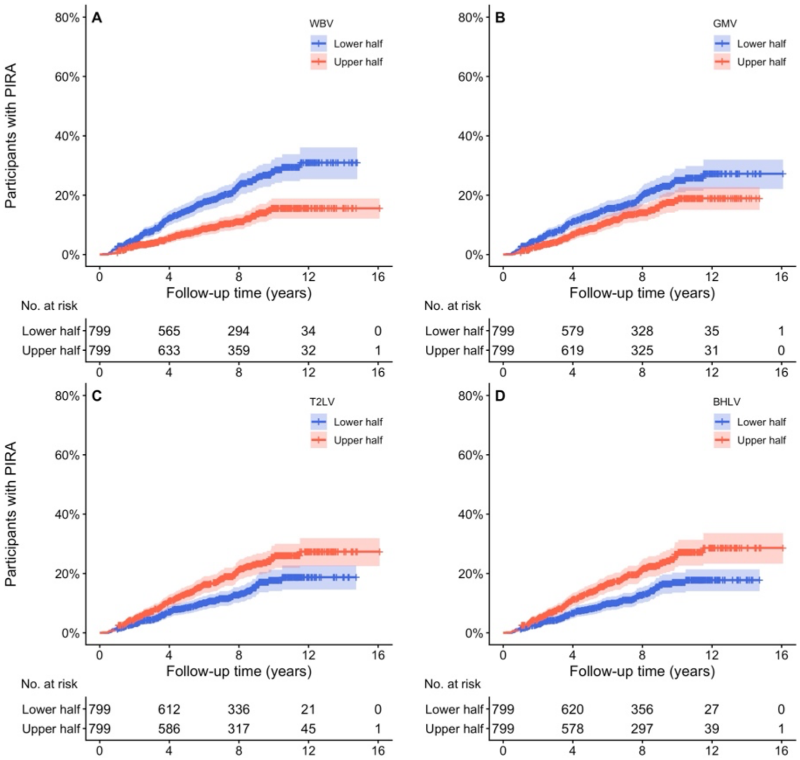

The red curve represents the upper half and the blue curve represents the lower half of the cohort (median split was performed) for the associated brain MRI volumetric measurement, with the lighter shade reflecting the 95% CI. Abbreviations: BHLV: black hole lesion volume; GMV: normalised grey matter volume; PIRA: progression independent of relapse activity; T2LV: T2 lesion volume; WBV: normalised whole brain volume.

Supplementary Figure 5. Survival curves for time to CDI for baseline GMV (A), WMV (B), T2LV (C) and BHLV (D)

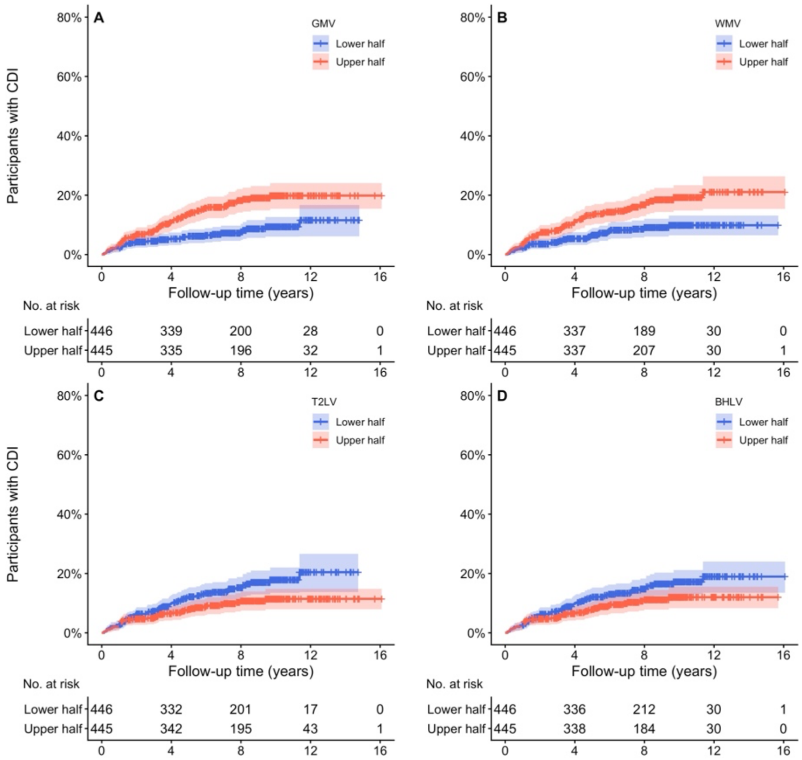

The red curve represents the upper half and the blue curve represents the lower half of the cohort (median split was performed) for the associated brain MRI volumetric measurement, with the lighter shade reflecting the 95% CI. Abbreviations: BHLV: black hole lesion volume; CDI: confirmed disability improvement; GMV: normalised grey matter volume; T2LV: T2 lesion volume; WMV: normalised white matter volume.

Supplementary References

1. Jain S, Sima DM, Ribbens A, Cambron M, Maertens A, Van Hecke W, De Mey J, Barkhof F, Steenwijk MD, Daams M, Maes F, Van Huffel S, Vrenken H, Smeets D (2015) Automatic segmentation and volumetry of multiple sclerosis brain lesions from MR images. Neuroimage Clin 8:367-375. https://doi.org/10.1016/j.nicl.2015.05.003
2. Rakić M, Vercruyssen S, Van Eyndhoven S, de la Rosa E, Jain S, Van Huffel S, Maes F, Smeets D, Sima DM (2021) icobrain ms 5.1: Combining unsupervised and supervised approaches for improving the detection of multiple sclerosis lesions. Neuroimage Clin 31:102707. https://doi.org/10.1016/j.nicl.2021.102707
3. Simarro J, Meyer MI, Van Eyndhoven S, Phan TV, Billiet T, Sima DM, Ortibus E (2024) A deep learning model for brain segmentation across pediatric and adult populations. Scientific Reports 14:11735. https://doi.org/10.1038/s41598-024-61798-6
